# Phylogenomic Analysis Identifies Gene Gains That Define *Salmonella enterica* Subspecies I

**DOI:** 10.1371/journal.pone.0076821

**Published:** 2013-10-28

**Authors:** E. Kurt Lienau, Jeffrey M. Blazar, Charles Wang, Eric W. Brown, Robert Stones, Steven Musser, Marc W. Allard

**Affiliations:** 1 Office of the Center Director, Center for Food Safety and Applied Nutrition, Food and Drug Administration, College Park, Maryland, United States of America; 2 Evolution Industries LLC, Frederick, Maryland, United States of America; 3 Department of Biology, University of Maryland, College Park, Maryland, United States of America; 4 The Food and Environment Research Agency, Sand Hutton, York, United Kingdom; Columbia University, United States of America

## Abstract

Comparative methods for analyzing whole genome sequence (WGS) data enable us to assess the genetic information available for reconstructing the evolutionary history of pathogens. We used the comparative approach to determine diagnostic genes for *Salmonella enterica* subspecies I. *S. enterica* subsp. I strains are known to infect warm-blooded organisms regularly while its close relatives tend to infect only cold-blooded organisms. We found 71 genes gained by the common ancestor of *Salmonella enterica* subspecies I and not subsequently lost by any member of this subspecies sequenced to date. These genes included many putative functional phenotypes. Twenty-seven of these genes are found only in *Salmonella enterica* subspecies I; we designed primers to test these genes for use as diagnostic sequence targets and data mined the NCBI Sequence Read Archive (SRA) database for draft genomes which carried these genes. We found that the sequence specificity and variability of these amplicons can be used to detect and discriminate among 317 different serovars and strains of *Salmonella enterica* subspecies I.

## Introduction

Recently, we applied whole genome phylogenetic analysis to the epidemiological trace-back of an outbreak of Salmonellosis [1]. However, analyses of this type can only give information about past outbreaks, and cannot prevent outbreaks from happening in the first place. In order to prevent outbreaks, we must be able to rapidly identify tainted foods before they come to market.

Some researchers have questioned whether it is possible to reconstruct an accurate evolutionary history of bacteria, given ongoing debates about the influence of horizontal gene transfer [2]–[9]. However, we believe that a phylogenetic analysis of whole genome sequence (WGS) data can solve these problems and account for HGT. In fact, using a whole genome tree of life hypothesis, we were recently able to corroborate the hypothesis that there is a vertical history of life for bacteria [8]. We expect these techniques will enable us to better understand the genomic evolutionary history of finer scale taxonomic classes of bacteria, including serovars of S. enterica subspecies I. As a step toward this goal, we have applied the comparative method of WGS phylogenetic analysis to discover diagnostic biomarkers [2] capable of identifying and discriminating among forms of *Salmonella enterica* (*S. enterica*). We suggest that phylogenetic analysis of WGS data can provide a solution to the problem of effective detection and identification of *S. enterica* serovars and some strains.

### The *Salmonella enterica* subspecies I

*Salmonella* infection is currently the most common foodborne illness in the United States (US), resulting in thousands of infections per year. These rates have not declined in over a decade, demonstrating the high fitness level of *S. enterica*. To reduce the human and financial costs of this pathogen, it is imperative to quickly and cheaply detect Salmonella contamination before it enters the food distribution system [10], [11].

The current classification of the genus *Salmonella* divides it into two species: *Salmonella bongori* and *Salmonella enterica* [12]. Baumler [13] suggested that a gain of the genetic elements *fim*, the *Salmonella* Pathogenicity Island 1 (SPI1), and *lpf*, are responsible for the ability of this genus to invade intestinal epithelial cells. In the same study, Baumler [13] went on to postulate that the evolutionary transition from the common ancestor of *S. bongori* and *S. enterica* to *S. enterica* occurred, in part, by the acquisition of SPI1, and that the divergence of *S. enterica* subspecies I from the other subspecies is due to the acquisition of several genes by subspecies I, and loss of the *lpf* operon by subspecies II, III, IV, and VI. Later, Baumler et al. [14] developed the hypothesis that the complex lymphoid systems of mammals and some bird species drove the evolution of virulence among all of the members of subspecies I. Later research from the same group reported that *invA* dependent SPI1 is responsible for the ability of non-typhoidal *Salmonella* to enter gut lymphoid systems [15].

Several approaches have been used to classify the serovars within *Salmonella enterica* subspecies I and some of the perceived disagreements among researchers may be attributable to differences in methodology. For example, one recent study showed that gene presence-absence data from DNA microarray analyses produced an un-weighted pairwise-distance tree that clusters most serovars together; however, multi-locus-sequence-typing (MLST) analysis showed more variability [16]. One study aimed at classifying serovars within *S. enterica* subspecies I using WGS information concluded that there is little correspondence of serotype with evolutionary history [17], although this analysis did not address any possible HGT. Another analysis explored gene gains in different subspecies of *S. enterica* from a functional point of view, noting abundant recombination events between lineages [18]. Another recent analyses with draft and complete genome sequences using Ribosomal 16s and weighted gene presence-absence matrices came to different conclusions based on the data type and weighting scheme used to correlate serotype and genomic evolutionary history [19]. An MLST and whole genome alignment analysis, using serotypes of both *S. bongori* and *S. enterica* that rooted the genus with *S. enterica* arizonae, found that serovars of *S. enterica* and *S. bongori* underwent HGT from other species [20]. Another *Salmonella* population genetics study, that sequenced 146 regions of 2 to 2.5 kb for 114 strains of *Salmonella enterica*, found there was significant homologous recombination in the species. Each of these analyses has provided a wealth of information that furthers our understanding of the evolutionary history and function of these important pathogens.

In the current project, we have used different draft genome sequences, in conjunction with complete genome sequences, to further test the evolutionary relationships within *Salmonella enterica* subspecies I to derive a better-corroborated history of these foodborne pathogens (Table 1). As draft genome data are only able to describe gene sequences that are present in, but not those absent from, a genome, we focused our analyses on those genes that were present in all samples used in our phylogenetic analysis.

**Table 1:** Genome sequences used in this analysis.

SRA NCBI	PRoject #	Taxids	Full Name
SRX129176	PRJNA41263	685040	Salmonella enterica subsp. enterica serovar Gallinarum str. 9184
SRX129211	PRJNA41461	687914	Salmonella enterica subsp. enterica serovar Rissen str. 150
SRX129213	PRJNA41463	687915	Salmonella enterica subsp. enterica serovar Dublin str. SL1438
SRX129216	PRJNA41465	687860	Salmonella enterica subsp. enterica serovar Dublin str. HWS51
SRX129219	PRJNA41915	696870	Salmonella enterica subsp. enterica serovar Enteritidis str. SE8a
SRX129220	PRJNA41917	696864	Salmonella enterica subsp. enterica serovar Enteritidis str. 20037
SRX129221	PRJNA41919	696865	Salmonella enterica subsp. enterica serovar Enteritidis str. SE10
SRX129224	PRJNA41921	696869	Salmonella enterica subsp. enterica serovar Enteritidis str. 436
SRX129225	PRJNA41929	696867	Salmonella enterica subsp. enterica serovar Enteritidis str. 18569
SRX129226	PRJNA41931	696866	Salmonella enterica subsp. enterica serovar Enteritidis str. 13–1
SRX129229	PRJNA41933	696868	Salmonella enterica subsp. enterica serovar Enteritidis str. PT23
SRX129230	PRJNA50691	866914	Salmonella enterica subsp. enterica serovar 4, [5],12:i:- str. 08–1700
SRX129231	PRJNA50693	866915	Salmonella enterica subsp. enterica serovar 4, [5],12:i:- str. 08–1739
SRX129232	PRJNA50695	866919	Salmonella enterica subsp. enterica serovar Kentucky str. 29439
SRX129233	PRJNA53275	891424	Salmonella enterica subsp. enterica serovar Typhimurium str. CDC_2009K1153
SRX129234	PRJNA53267	891420	Salmonella enterica subsp. enterica serovar Javiana str. ATCC BAA-1593
SRX129235	PRJNA46537	745016	Salmonella enterica subsp. enterica serovar Montevideo str. 316111868
SRX101636	PRJNA46539	745017	Salmonella enterica subsp. enterica serovar Montevideo str. 495297–1
SRX101642	PRJNA46541	745018	Salmonella enterica subsp. enterica serovar Montevideo str. 495297–3
SRX101643	PRJNA46543	745019	Salmonella enterica subsp. enterica serovar Montevideo str. 495297–4
SRX101644	PRJNA46545	745020	Salmonella enterica subsp. enterica serovar Montevideo str. 515920–1
SRX101645	PRJNA46547	745021	Salmonella enterica subsp. enterica serovar Montevideo str. 515920–2
SRX101646	PRJNA46549	745022	Salmonella enterica subsp. enterica serovar Montevideo str. 531954
SRX101649	PRJNA46907	749951	Salmonella enterica subsp. enterica serovar Montevideo str. CASC_09SCPH15965
SRX105725	PRJNA46911	749952	Salmonella enterica subsp. enterica serovar Montevideo str. SaRB31
SRX105759	PRJNA46913	749953	Salmonella enterica subsp. enterica serovar Montevideo str. ATCC BAA710
SRX105760	PRJNA46915	749948	Salmonella enterica subsp. enterica serovar Montevideo str. LQC 10
SRX105761	PRJNA46917	749947	Salmonella enterica subsp. enterica serovar Montevideo str. SaRB30
SRX129236	PRJNA46931	749930	Salmonella enterica subsp. enterica serovar Tennessee str. 4535
SRX129237	PRJNA49407	789642	Salmonella enterica subsp. enterica serovar Newport str. CVM 35185
SRX129238	PRJNA49409	789643	Salmonella enterica subsp. enterica serovar Newport str. CVM 35199
SRX129239	PRJNA49411	789644	Salmonella enterica subsp. enterica serovar Newport str. CVM 21539
SRX129240	PRJNA49625	796735	Salmonella enterica subsp. enterica serovar Newport str. CVM 19567
SRX129241	PRJNA51207	99287	Salmonella enterica subsp. enterica serovar Typhimurium str. LT2
	PRJEA28309	496064	Salmonella enterica subsp. enterica serovar Typhi str. E98–3139
	PRJEA28303	496067	Salmonella enterica subsp. enterica serovar Typhi str. J185
	PRJNA371	209261	Salmonella enterica subsp. enterica serovar Typhi Ty2
	PRJEA28295	497974	Salmonella enterica subsp. enterica serovar Typhi str. E02–1180
	PRJEA30943	554290	Salmonella enterica subsp. enterica serovar Paratyphi A str. AKU_12601
	PRJNA13086	295319	Salmonella enterica subsp. enterica serovar Paratyphi A str. ATCC 9150
	PRJNA20993	476213	Salmonella enterica subsp. enterica serovar Paratyphi C strain RKS4594
	PRJNA9618	321314	Salmonella enterica subsp. enterica serovar Choleraesuis str. SC-B67
	PRJNA20595	465517	Salmonella enterica subsp. enterica serovar Virchow str. SL491
	PRJNA19465	440534	Salmonella enterica subsp. enterica serovar 4, [5],12:i:- str. CVM 23701
	PRJNA19461	439846	Salmonella enterica subsp. enterica serovar Saintpaul str. SaRA23
	PRJNA20065	454164	Salmonella enterica subsp. enterica serovar Heidelberg str. SL486
	PRJNA20045	454169	Salmonella enterica subsp. enterica serovar Heidelberg str. SL476
	PRJNA20593	465516	Salmonella enterica subsp. enterica serovar Hadar str. RI_05P066
	PRJNA27803	1016998	Salmonella enterica subsp. enterica serovar Paratyphi B str. SPB7
	PRJNA19463	439847	Salmonella enterica subsp. enterica serovar Saintpaul str. SaRA29
	PRJEA30687	550537	Salmonella enterica subsp. enterica serovar Enteritidis str. P125109
	PRJNA19467	439851	Salmonella enterica subsp. enterica serovar Dublin str. CT_02021853
	PRJNA30831	573395	Salmonella enterica subsp. enterica serovar Tennessee str. CDC 07–0191
	PRJNA19457	439842	Salmonella enterica subsp. enterica serovar Kentucky str. CVM 29188
	PRJNA20069	454231	Salmonella enterica subsp. enterica serovar Kentucky str. CDC 191
	PRJNA20063	454166	Salmonella enterica subsp. enterica serovar Agona str. SL483
	PRJNA20591	465518	Salmonella enterica subsp. enterica serovar Weltevreden str. HI_N05–537
	PRJNA20049	454167	Salmonella enterica subsp. enterica serovar Javiana str. GA_MM04042433
	PRJNA20071	454165	Salmonella enterica subsp. enterica serovar Schwarzengrund str. SL480
	PRJNA19459	439843	Salmonella enterica subsp. enterica serovar Schwarzengrund str. CVM19633
	PRJNA20047	454168	Salmonella enterica subsp. enterica serovar Newport str. SL317
	PRJNA18747	423368	Salmonella enterica subsp. enterica serovar Newport str. SL254
	PRJEA30689	550538	Salmonella enterica subsp. enterica serovar Gallinarum str. AM933173
	PRJEA70155	218493	Salmonella bongori NCTC 12419
	PRJEA13030	41514	Salmonella enterica subsp. arizonae serovar 62:z4, z23:–

## Results/Discussion

### The *Salmonella enterica* subspecies I

**Figure 1:**
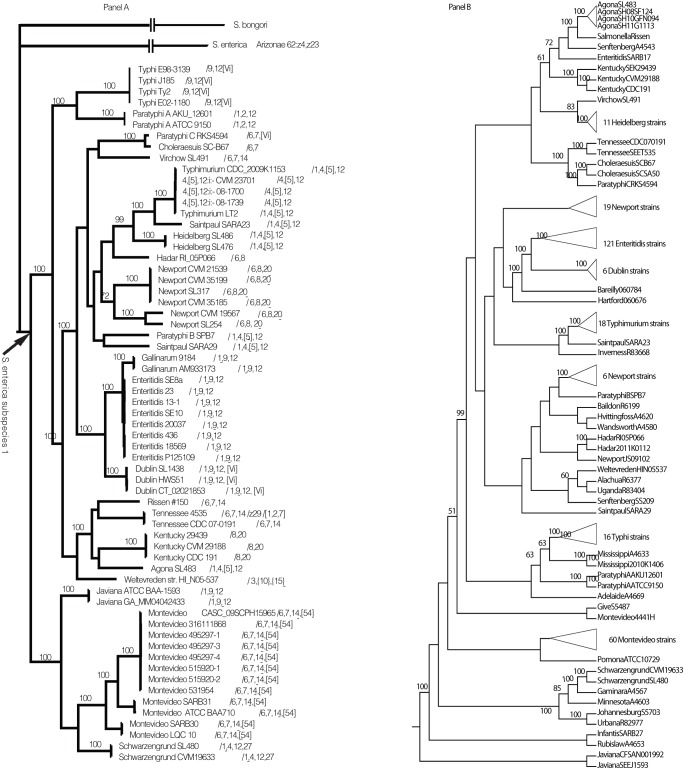
The strict consensus tree of the 321 most-parsimonious trees based on the nucleotide substitutions for 937 genes (A). This tree postulates an evolutionary hypothesis that classifies most operational taxonomic units as monophyletic within the same O-antigen serovar but shows that the 1,4,5,12:i:- O-antigen complex is polyphyletic. Bootstrap percentages of 50% and over are listed above nodes, except those leading to taxa that are grouped together by branch lengths too short to be visible on the figure. The *Salmonella enterica* subspecies I clade is indicated with an arrow. The branch lengths leading to the out-groups *S. bongori* and *S. enterica* subspecies arizonae are not drawn to scale as denoted by the broken branches leading to these taxa. The rest of the branch lengths are drawn to scale. Panel (B) shows the majority rule bootstrap consensus tree using only the information contained in the 27 predicted PCR amplicon sequences of 317 strains of *S. enterica* subspecies I. It is rooted with the *Salmonella enterica* subspecies I serotype Javiana clade. Clades containing only members of the same serovars are collapsed into a single schematic. Many of the schematic-depicted clades often had internal resolution (See Figure S1).

We used gene presence-absence data and the phylogenetic methods of Lienau et al. [21], [22] as heuristic searches to empirically define the *Salmonella enterica* subspecies I homologous genes. Briefly, these searches define gene similarity thresholds and select the threshold resulting in the most resolved and consistent gene presence-absence phylogeny that also provides the most consistent character statements as measured by the combined corroboration metric (CCM) [21]. Our phylogenetic analysis and homology search showed that a similarity value of 70% yielded the most congruent and resolved gene presence-absence phylogeny. We only considered open reading frames (ORFs) that are longer than 120 base pairs and matched at least one other ORF across more than 80% of the nucleotide sequence. The heuristic analysis identified 937 genes common to all organisms in our analysis; this is about half the number found by Jacobsen et al. [19], due to the stringency with which we defined our homologous groups. We used these commonly-held genes to create a multi-locus DNA sequence evolution phylogenetic matrix of 937 genes that contained 204,753 total characters. Out of this number, 82,147 characters were constant, 74,345 were variable characters and parsimony-uninformative, and 48,261 characters were parsimony-informative. Parsimony analysis of this matrix produced 321 most parsimonious trees with a score of 201,886 and CI of 0.653; the strict consensus tree gave a normalized consensus fork component information index of 0.887. We rooted this strict consensus tree with the *S. bongori* genome and chose to depict this rooting as a polyphyletic relationship. This root placed *S. enterica arizonae* at the base of the *S. enterica* subspecies I lineage. We used broken branches, as seen at the top of Figure 1, to denote that *S. bongori* and *S. enterica arizonae* had long internal branch lengths compared to the *S. enterica* subspecies I, a result shared with Fookes, et al. [20].

The strict consensus tree postulates an evolutionary hypothesis classifying most operational taxonomic units (OTUs) that belong to the same O-antigen serovar as monophyletic groups (Figure 1A). There is one notable exception. The O antigen class *S. enterica subsp. enterica* serovar 1,4,5,12:i:- (*S.* 1,4,5,12:i:-) is polyphyletic, perhaps due to reticulate evolution via HGT, loss of some antigenic components, or convergent evolution of the same O antigen moiety in distantly-related groups. In figure 1A, the first clade of organisms that shows the *S.* 1,4,5,12:i:- O antigen phenotype is the *S*. Heidelberg, *S*. Saintpaul SARA23, *S.* Typhimurium and *S.* 1,4,5,12:i:- clade. The evolution of the O antigen phenotype in this group could be satisfactorily explained by the loss of the “1” characteristic of the somatic (O) antigen phenotype in the *S,* 1,4,5,12:i:- taxa after a gain of the *S.* 1,4,5,12:i:- by the common ancestor of the clade. The *S.* 1,4,5,12:i:- phenotype is also exhibited by the *S*. Paratyphi B, *S*. Saintpaul SARA29 clade (shown in bold toward the middle of the tree in panel A of Figure 1) and by *S.* Agona (shown in bold toward the bottom of the tree). It is tempting to speculate that the *S.* 1,4,5,12:i:- phenotype came into these taxa via one or more HGT events conferring genes responsible for generating the somatic antigens of *S. enterica*.

This tree also resolves the *S. enterica* subspecies I relationships for 12 of 35 strains of *S.* Montevideo [1] and places *S.* Javiana and *S.* Schwarzengrund at the base of the monophyletic *S.* Montevideo group. *S.* Enteritidis is monophyletic and clusters with a monophyletic *S.* Galinarum and a monophyletic *S.* Dublin. In contrast to the findings of Achtman, et al. [16], *S.* Kentucky and *S*. Tennessee appear to be monophyletic. These discrepancies may be due to the different phylogenetic and/or sampling methods and isolates used in these analyses.

### Genes gained at the base of the *Salmonella enterica* subspecies I

We then mapped the gene presence-absence data onto the core gene phylogeny to identify genes gained by *S. enterica* subspecies I. Previous researchers have identified from 216 to 249 genes gained by the *S. enterica* subspecies I. [13], [23]–[25] We examined all the genes gained by the common ancestor of all *S. enterica* subspecies I in our analysis. We identified 377 total genes gained. Of these, 71 genes were gained once and not lost by any members of *S. enterica* subspecies I included in this analysis (Table 2, see methods).

**Table 2:** *S. enterica* subspecies I genes with similarity to other species.

Putative protein product function	Organisms with similar DNA sequences
**cytoplasmic protein citx**	Escherichia coli
**DNA-binding transcriptional regulator DsdC**	Citrobacter koseri, Citrobacter rodentium, Klebsiella oxytoca, Klebsiella oxytoca, Klebsiella pneumoniae, Escherichia coli Klebsiella variicola, Klebsiella pneumoniae subsp. pneumonia, Enterobacter aerogenes KCTC 2190, Escherichia fergusonii, Edwardsiella tarda, Enterobacter asburiae, Enterobacter cloacae, Serratia proteamaculans, Aeromonas salmonicida subsp. salmonicida, Aeromonas hydrophila subsp. hydrophila
**sugar-binding domain protein**	Shigella flexneri, Escherichia coli, Escherichia fergusonii, Klebsiella pneumoniae subsp. pneumoniae, Photorhabdus asymbiotica, Klebsiella pneumoniae, Yersinia pseudotuberculosis, Klebsiella variicola At-22, Yersinia pestis
**Propanediol utilization: propanol dehydrogenase pdxA**	Klebsiella oxytoca, Citrobacter rodentium, Enterobacter cloacae
**oxalacetate decarboxylase subunit beta**	Klebsiella oxytoca, Klebsiella pneumoniae subsp. pneumoniae, Klebsiella pneumoniae, Klebsiella variicola, Enterobacter aerogenes, Marinobacter hydrocarbonoclasticus, Marinobacter adhaerens HP15, Pseudomonas mendocina, Marinobacter aquaeolei
**hexulose-6-phosphate isomerase**	Klebsiella oxytoca, Klebsiella pneumoniae, Klebsiella pneumoniae, Citrobacter koseri, Escherichia coli, Shigella flexneri, Enterobacter cloacae
**propanediol dehydratase reactivation protein**	Klebsiella oxytoca, Klebsiella pneumoniae, Citrobacter rodentium
**lipoprotein**	Escherichia fergusonii, Escherichia coli, Shigella sonnei, Shigella boydii,
**aldolase**	Escherichia coli, Shigella flexneri, Xenorhabdus bovienii, Shigella dysenteriae, Xenorhabdus nematophila, Yersinia pseudotuberculosis, Yersinia pestis, Photorhabdus luminescens subsp. laumondii, Photorhabdus asymbiotica,
**citrate lyase beta chain**	Enterobacter cloacae subsp. Cloacae
**4-hydroxythreonine-4-phosphate dehydrogenase**	Citrobacter rodentium, Klebsiella oxytoca, Dickeya dadantii, Dickeya zeae, Pectobacterium carotovorum subsp. carotovorum, Pectobacterium carotovorum subsp. carotovorum, Erwinia carotovora subsp. atroseptica, Pantoea vagans, Pantoea sp. At-9b, Pectobacterium sp. SCC3193, Enterobacter aerogenes, Pectobacterium wasabiae, Escherichia coli, Erwinia billingiae, Escherichia coli, Enterobacter cloacae subsp. cloacae, Enterobacter asburiae, Pantoea ananatis, Enterobacter cloacae subsp. dissolvens, Klebsiella variicola, Klebsiella pneumoniae, Escherichia blattae, Klebsiella pneumoniae, Klebsiella pneumoniae subsp. pneumoniae
**peptide chain release factor**	Escherichia fergusonii, Escherichia coli, Shigella sonnei, Shigella boydii, Shigella flexneri
**inner membrane protein YqiK**	Citrobacter rodentium, Escherichia fergusonii, Shigella sonnei, Shigella boydii, Shigella dysenteriae
**inner membrane transport protein YdhP**	Klebsiella pneumoniae subsp. pneumoniae, Enterobacter cloacae subsp. dissolvens, Klebsiella variicola, Klebsiella pneumoniae, Enterobacter asburiae, Enterobacter cloacae subsp. cloacae, Enterobacter aerogenes, Enterobacter cloacae, Dickeya dadantii 3937, Klebsiella oxytoca, Escherichia blattae, Serratia sp. AS13, Serratia sp. AS12, Serratia plymuthica, Serratia proteamaculans, Rahnella aquatilis HX2, Rahnella sp. Y9602, Pantoea sp. At-9b,, Yersinia enterocolitica subsp. palearctica, Yersinia enterocolitica, Yersinia enterocolitica subsp. enterocolitica, Aeromonas veronii, Polaromonas naphthalenivorans, Collimonas fungivorans Ter331, Variovorax paradoxus, Ralstonia solanacearum, Aeromonas salmonicida subsp. salmonicida, Aeromonas hydrophila subsp. hydrophila, Pseudomonas protegens, Azoarcus sp. BH72, Ferrimonas balearica, Pseudomonas fluorescens, Chromobacterium violaceum, Shewanella amazonensis, Stenotrophomonas maltophilia R551–3, Cupriavidus necator, Stenotrophomonas maltophilia, Acidovorax avenae subsp. avenae, Halomonas elongata, Pseudogulbenkiania sp. NH8B DNA, Pseudomonas stutzeri, Acidovorax sp. KKS102, Glaciecola sp. 4H-3–7+YE-5, Acidovorax citrulli
**tartrate dehydratase subunit beta**	Klebsiella oxytoca, Enterobacter aerogenes, Escherichia fergusonii, Klebsiella pneumoniae subsp. Pneumoniae
**3-keto-L-gulonate-6-phosphate decarboxylase**	Klebsiella oxytoca, Citrobacter koseri, Klebsiella pneumoniae, Escherichia coli, Shigella flexneri, Enterobacter cloacae, Enterobacter cloacae subsp. dissolvens, Enterobacter asburiae, Enterobacter cloacae subsp. cloacae, Gryllus bimaculatus, Serratia proteamaculans, Serratia sp. AS13, Serratia sp. AS12, Serratia plymuthica, Pantoea sp. At-9b, Pectobacterium sp. SCC3193, Pectobacterium carotovorum subsp. carotovorum PC1, Pantoea ananatis
**propanediol utilization protein PduJ**	Klebsiella oxytoca, Citrobacter rodentium, Citrobacter freundii, Enterobacter cloacae, Citrobacter koseri, Uncultured bacterium, Escherichia fergusonii,
**acetyltransferase, gnat family**	Enterobacter cloacae subsp. cloacae, Citrobacter koseri, Citrobacter rodentium, Klebsiella oxytoca, Klebsiella pneumoniae subsp. pneumoniae, Klebsiella pneumoniae, Enterobacter cloacae subsp. cloacae, Enterobacter cloacae subsp. dissolvens, Klebsiella variicola, Enterobacter aerogenes, Escherichia coli, Shigella flexneri, Escherichia fergusonii, Escherichia coli, Shigella boydii
**inner membrane protein ybcI**	Enterobacter cloacae subsp. cloacae, Enterobacter cloacae, Escherichia fergusonii, Enterobacter cloacae subsp. dissolvens SDM, Escherichia coli, Shigella boydii, Shigella sonnei, Shigella dysenteriae
**ABC transport protein, solute-binding component**	Escherichia coli, Shigella flexneri, Klebsiella variicola, Escherichia fergusonii, Klebsiella pneumoniae, Enterobacter aerogenes, Yersinia enterocolitica subsp. enterocolitica, Rahnella aquatilis, Yersinia enterocolitica, Yersinia enterocolitica subsp. palearctica, Rahnella aquatilis, Rahnella sp. Y9602,, Yersinia pestis
**outer membrane protein**	Escherichia fergusonii
**GumN Family transcriptional regulator**	Klebsiella pneumoniae subsp. pneumoniae, Enterobacter aerogenes, Klebsiella variicola At-22, Klebsiella pneumoniae, Klebsiella pneumoniae, Klebsiella oxytoca Enterobacter cloacae
**GntR Family transcriptional regulator**	Klebsiella oxytoca, Enterobacter aerogenes, Klebsiella variicola, Klebsiella pneumoniae, Klebsiella pneumoniae subsp. pneumoniae,
**oxalacetate decarboxylase, subunit gamma**	Klebsiella pneumoniae subsp. pneumoniae, Klebsiella pneumoniae, Klebsiella variicola, Klebsiella oxytoca, Enterobacter aerogenes, Escherichia fergusonii
**LsrG**	Escherichia coli, Yersinia enterocolitica, Xenorhabdus nematophila, Photorhabdus luminescens subsp. laumondii, Yersinia pestis, Serratia sp. AS13, Serratia sp. AS12, Serratia plymuthica AS9, Yersinia pestis biovar Medievalis ,
**fimbrial chaperone protein**	Klebsiella pneumoniae subsp. pneumoniae
**citrate-sodium symport**	Klebsiella oxytoc, Klebsiella pneumoniae subsp. pneumoniae, Klebsiella pneumoniae, Enterobacter aerogenes, Klebsiella variicola, Escherichia fergusonii, Vibrio sp. EJY3, Vibrio cholerae, Photobacterium profundum
**outer membrane protein**	Escherichia fergusonii
**glycosyl hydrolase**	Escherichia coli NA114, Escherichia coli, Rahnella aquatilis HX2 plasmid PRA1 Rahnella sp. Y9602 plasmid pRAHAQ01
**aspartate alpha-decarboxylase**	Citrobacter koseri ATCC BAA-895, Enterobacter cloacae EcWSU1, Enterobacter asburie LF7a, Klebsiella variicola At-22, Klebsiella pneumoniae 342, Klebsiella pneumoniae subsp. pneumoniae HS11286,
**LSRK**	Escherichia coli, Escherichia fergusonii, Serratia sp. AS13, Serratia sp. AS12, Serratia plymuthica AS9, complete genome 888 933 91% 0.0 93% Xenorhabdus bovienii, ersinia pseudotuberculosis, Yersinia pestis, Yersinia pseudotuberculosis, Rahnella aquatilis, Serratia proteamaculans, Yersinia enterocolitica subsp. enterocolitica 8, Photorhabdus asymbiotica, Photorhabdus asymbiotica subsp. asymbiotica, Yersinia enterocolitica, Photorhabdus luminescens subsp. laumondii, Rahnella aquatilis HX2, Xenorhabdus nematophila

We then identified ORFs at the nucleotide level that are both specific to and able to discriminate among the genetically known serovars of *Salmonella enterica* subspecies I included in this study. We used nucleotide blasts to compare members of these genes against the NCBI non-redundant (nr) nucleotide database. Thirty-one gene sequences had a BLASTn total score of greater than or equal to 200 vs. non-*Salmonella* organisms (Table 3). We also retrieved 13 significant hits using the default parameters of megaBLAST to *S. enterica houtenae*, (subspecies IV) of *Salmonella enterica* in the SRA http://www.ncbi.nlm.nih.gov/Traces/sra).

**Table 3:** Primers, product length, locus ID and putative function of target sequences for *S. enterica*.

L Sequence	R Sequence		P Size	gene name	Accession Number
ATCGTTGAAGACGAACCGAT	GCCTTGTTCCAGATAGCGTC		585	CitB – citrase metablism	CP001120.1
GGAATCCAGCGATGTGATGAT	GCACTCCATTGAATTTCCGT		252	Lyase subunit gamma	AM933172.1
GGATCATGCCAGCGATTATT	GTTACTGTTTGTCCGCCGAT		573	StbD fimbrial protein usher	CP000886.1
CTTGGCTTTAACCGCATGTA	TAACGAGGGCAACGATAAAC		330	CycH cytochrome c biogenesis	CP001120.1
ATTTTGCCCCAGCAGTTATG	CATTCAGTGGTTCGTTGGTG		515	No database matches	CP001144.1
GTGCCTGTAAGCACTCAATCTT	GGCAAGATAATCGACGCCTA		186	Pathogenicity island protein	AE014613.1
ATGTCGATCGGCAGAAAATC	TGAATCAATTGCGTCAGGAG		559	2-dehydropantoate 2-reductase	CP002614.1
CACTCATGGCGCAATATCAC	CGCCAGAATCATAAACAGCA		566	Putative permease MFS	CP001144.1
GCTCGACAATATCAAAGGCG	ATCGCCAGATCCCATACTGA		447	Putative cytoplasmic protein	CP001127.1
CGTTCGTTTTCTTTATCGGC	TCAGCACCTTGTCTCCTTTT		519	Inner membrane protein	FR775236.1
TGTTTTCTGTGTTGAAGCCG	AACGTCATTAAAACCGCACC		574	Transcriptional regulator	AM933172.1
CTAAATTCAAAAGCCCTGCC	CATACCAGCACCTTTAGCCC		310	DNA-binding protein	AM933173.1
AGAATTTTTGGTGGCCTCG	TGTCATTCCACACGTCCATC		323	NTF2_like	CP001113.1
GGGTGACGACCTTTCAAAAA	ATGCTGTCTGCATTGGTCTG		502	Hypothetical protein	AM933172.1
CAAACATGAACGTTCGCATC	GCGGTAGCTTCTTTGACGAC		502	FGGY_N super family [cl09121],	CP001138.1
AGGCCGAAAAGAAAATCCAC	ACAGTCGTCTGGCGCTATCT		534	MOSC	CP001120.1
CACGTTACGCTGGTTGAAAT	TTGTCTTGTGAAAAGCGACG		199	ABM	AM933172.1
GTGCGGTTTTATCGGTTGTC	TGGTGTCACAGCCTAGATCG		512	Aminoimidazole riboside kinase Fructokinases (FRKs)	CP001144.1
AAACGCCATTCAGCAATCTC	TATTTGCGGTAGGTTGCCTT		566	Uncharacterized protein conserved in bacteria [Function unknown]	AM933172.1
CCATTATCGTTTCTGCCGTT	CCCACAGGCCAAACTCTTTA		540	PBP1_LsrB_Quorum_Sensing	AM933172.1
TGCAGGTTGATAAAACGAGC	CGGGAAAGAAGCTAAGATCG		468	DNA-binding domain of the GntR family of transcriptional regulators	CP001127.1
CGGTCCGTAAAGTTCTCGAC	CGGTATCAATGTGCATTTCG		543	Propanediol utilisation protein PduL	CP001138.1
TCGGTTCATGCATTTACGTC	TACCGTTGTGAGCGGTAAAG		313	Undecaprenyl-phosphate alpha-N- acetylglucosaminyltransferase	CP000886.1
TCATGTTGCTGCAGGAGATT	CGGTAACGCTCTTTTATGTCG		555	Putative cytoplasmic protein	CP001138.1
GTAGCGTCGCTTTCTGAACC	GCCTCCAGTCGTGCATTATT		503	Putative methyl-accepting chemotaxis protein	CP001144.1
ATACCGCTTTGGCGATAATG	TGGCTGTCATTTTCCAGATG		297	Allantoin catabolism protein	FM200053.1
CTGGAAGGCGAGAAACAGTC	CATGGTGGCGACAGTATCAG		543	ADP-ribosylglycohydrolase superfamily	CP001127.1
S. enterica subspecies 1 and houtenae					
CGGATGTCGCCTGTCTTATC	GCACGTTGTTCTGGCGTTAC	355		Pathogenicity island 2 effector protein SseG	CP002614.1
TTATGCCGCAGAATACTGGA	TATCAAGACGACTGATGCCG	245		Phage shock protein E	CP000857.1
CAACTACCTCGGCTATTCGC	GAAGAAAGCTTTGCCTGTGG	506		Fimbriae Y protein	CP001120.1
ACTATCAATATGGCAGCCCG	CATACGCGACAAGATAGCGA	509		PMT	CP001127.1
CTGGGCGCTGATGTTTTTAC	CCGGGTAACATCCTTGAAAA	363		Cytochrome_b_N	AM933172.1
GTATGCTGCGCCATCCTATT	GATGCTGAATATCTTCGCCG	135		Entericidin	CP000857.1
TATTTCATTCGCGCGGCTAC	GTAATCGCTCCAGCACATCC	501		AraC family	AM933172.1
ATGCGGTATGTGATTTCGGT	TCAGCCACGGGATTATAAGG	514		Molybdate ABC transporter periplasmic molybdate-binding protein	CP001113.1
CATCTCTGGGCGAAGTGAAT	ATTGATCAGGAAGGTCGCAT	443		FlmF putatuve fimbral protein	CP002614.1
TGAGTAAGCCACCGCTTTTC	GTACGGCTAACAAACCCGAC	355		Inner membrane protein	CP002614.1
CGAATAATAGCAACGGCGTC	GCAAACGTCGTGTTCGTTTT	143		YoaG conserved cytoplamsic	CP002614.1
AACGGCTGTGGTAAATCGAC	CTCCGCAGTAATCACCGTCT	570		ABC transporters	AE014613.1
CAGCGCTTGCGTCATTTAT	AGATCGCTGCGTGATTTCAG	160		Prokaryotic membrane lipoprotein	FM200053.1

This left 27 genes completely specific to *S. enterica* subspecies I and 31 genes with homologs that appear closely related to other non*-Salmonella* species. The functional classes of these gained genes were varied, indicating that the evolutionary advantage of gene gains to *S. enterica* subspecies I is not limited to a specific biological pathway (Tables 2 and 3). The relatively equal number of gene gains with similarity to non-*Salmonella* species (n = 31), compared to those with only weak similarity to other organisms (n = 27), may indicate that recent HGT from distantly related species as well as duplication and divergence within *Salmonella enterica* subspecies I may play a major role in the evolution of *S. enterica* subspecies I.

### Biomarkers to create improved detection tools for *Salmonella enterica* subspecies I

We also assessed the suitability of these gene sequences for use as biomarkers in a PCR and/or sequence detection system. The system was designed to quickly identify and/or discriminate among different serovars of *S. enterica* subspecies I. First, we tested the 27 *S. enterica* subspecies I-specific genes for utility as diagnostic biomarkers for *S. enterica* subspecies I. We designed PCR primers for all 27 biomarker candidates (Table 3, methods) and blasted all 27 sequences against members of *S. enterica* in the SRA [26]. These sequences showed significant similarity to 317 strains of *S. enterica* subspecies I, suggesting that these amplicons could be used as a gene presence-absence diagnostic test for *S. enterica* subspecies I. The primers were also tested in the laboratory on a limited set of isolates and amplicons were successfully generated as designed both for positive and negative expectations (data not provided).

We then tested the resolution capabilities of a PCR and sequencing-based test to discriminate among the 317 available strains of *S. enterica* subspecies I by doing phylogenetic analysis on the predicted PCR fragment sequences. We made 27 alignments for 317 *S. enterica* subspecies I serotypes and concatenated them into a MLST super-matrix composed of 12055 characters, 862 of which were parsimony-informative. We analyzed the matrix using the parsimony ratchet strategy. This analysis yielded 99 most parsimonious trees with scores 3720; the majority rule bootstrap consensus is shown in Figure 1B and the strict consensus is shown in Figure S1. While this tree is based on less character information and contains nearly 5 times the number of taxa than the tree derived from the 937 gene analysis, it is resolved enough to discriminate among most of the different serotypes and strains of *S enterica* subspecies I included in our analysis (Figure 1B).

In order to test whether we could increase the resolution of this MLST approach, we also selected gene sequence fragments for the 13 genes determined to be significantly similar to *S. enterica houtenae* in the SRA megaBLAST test. We designed and tested PCR primers to amplify these genes (Table 3). We included this additional genetic data to the 27 gene alignments for 317 *S. enterica* subspecies I serotypes and concatenated them into a MLST super-matrix composed of 319 taxa (including 2 *S. enterica houtenae* strains). This matrix had 16221 characters, 1361 of which are parsimony-informative. Phylogenetic analysis yielded 256 most parsimonious trees with scores 5820, the strict consensus of which is pictured in Figure S2. This tree is rooted with *S. enterica houtenae*. It is resolved enough to discriminate among all of the different serotypes and many strains of *S. enterica* subspecies I tested. This gene set is an improvement to the smaller MLST analysis that did not include the genes also present in *S. enterica houtenae* (Figure S2). These results indicate this set of PCR amplicons should be excellent candidate biomarkers for use in MLST and single nucleotide polymorphism (SNP) detection and diagnostic tools for *S. enterica* subspecies I serovars and some strains.

The first set of 27 genes and the PCR amplicons derived from them are sufficient for a gene presence-absence diagnostic test for *S. enterica* subspecies I. This set of amplicons can also be used to reliably determine most serotypes and some strains of *S. enterica* subspecies I in a PCR/SNP detection system. However, because some isolates did not resolve all serotypes into monophyletic clades when using the 27 gene set, it would be better to use the 40 gene set which includes the out-group *S.enterica houtenae* as a SNP-based MLST diagnostic system, since that method fully resolves all serotypes and many strains of *S enterica* subspecies I tested (Figure S2).

### Conclusion

Reconstructing the evolutionary history among lineages provides an approach for both identifying the qualities that make pathogens dangerous and detecting those organisms in settings where they pose potential threats to human health. We devised a scheme that would generate an evolutionary hypotheses to test which genes were unique among *Salmonella* to *S. enterica* subspecies I. Using the BLAST algorithm, we tested the origin of these gene sequences against the non-redundant nucleotide database and found that some genes were very similar to distantly related organisms, and that others were only weakly similar to distantly related species. We used these unique gene sequences to generate diagnostic biomarkers that can detect the presence and determine the serotype of *S. enterica* subspecies I. This method of identifying diagnostic characters for a clade of organisms provides a future framework to generate and test hypotheses about genetic variations that may be correlated with disease phenotypes.

## Materials and Methods

### Genome Sequences

We used Roche 454 sequencing technology to sequence 34 new *Salmonella enterica* draft genomes from various sources (Table 1). We assembled the shotgun sequenced genomes using Glimmer (http://cbcb.umd.edu/software/glimmer/) and had them annotated using the National Center for Biotechnology Information (NCBI) Prokaryotic Genome Automatic Annotation Pipeline (PGAAP) [27]. We also downloaded 30 publicly-available *Salmonella* species genome sequences from NCBI's GenBank, including *Salmonella bongori* (Accession NC_015761). This yielded a total of 71 *Salmonella enterica* genomes and one *Salmonella bongori* genome. The sequences reported in this paper have been deposited in the GenBank and SRA databases, with accession numbers listed in Table 1.

### Phylogenetic analysis

An empirical homology cluster search was performed per Lienau et al. [8] to determine the similarity value to generated the gene clusters that yielded the most congruent and best-resolved gene presence-absence phylogenetic tree. We tested similarity value thresholds for gene clustering of 60%, 70%, 80% and 90% at length limit of 120 bp and minimum match length of 80% using megaBLAST, as implemented in a computer program called PathGenome, currently being developed by the FDA and the Food and Environmental Research Agency (FERA). We generated gene presence-absence matrices for each of the similarity values tested and performed tree searches using the Phylogenetic Analysis using Parsimony and Other Methods (PAUP*) 4.10 b portable version [28] with a ratchet search of 9 iterations: 3 iterations each at perturbations of 15% 17% and 21%, respectively, using command files generated by Parsimony Analyses using PAUP* (PRAP) [29]. After establishing which genes in our study resulted in the gene presence-absence tree with the highest CCM score, we then aligned each of those gene sequences using MUSCLE and constructed a multi-locus DNA sequence evolution matrix [30]. We searched for the most optimal tree using the parsimony ratchet searches as described above. All characters were equally weighted. We did a bootstrap analysis on all phylogenetic matrices using PAUP*4.10 b portable version [28] at 100 replicates, holding a maximum of 1000 trees per replicate.

### Biomarker identification

We used the method of Lienau, 2012 [2] to identify likely candidate genes for use as diagnostic biomarkers. We defined the node of interest as the node that led to all of the *Salmonella enterica* subspecies I and then used accelerated transformation of parsimony character reconstruction to identify genes gained at that node. We then selected 71 of these gained genes that showed perfect consistency with the phylogenetic hypothesis as measured by the consistency index of Kluge and Farris [31]. We manually checked these genes for presence in all members of the ingroup (*S.enterica* subspecies I) and absence in the outgroup (*S. bongori* and *S. enterica arizonae*) to rule out symplesiomorphies. All 71 genes met these criteria. We then checked other organisms to see whether these genes were present (see next section).

### Blast to identify potential false positive markers and primer design

We took an example sequence from each of the 71 genes and blasted them against the nr nucleotide database at NCBI. Using a lower bound of 70% identity, we separate 40 genes with positive hits to only Salmonella, leaving aside the 31 genes that had hits to other organisms. We designed and tested primers to the conserved regions of the 40 *Salmonella-*only genes using MacVector with Assembler 11.0.2 via Primer Design (Primer3). We set the ideal amplicon length to between 500 and 600 base pairs. (Table 3). Using phylogenetic analysis (Figure S2), we further tested the potential utility of these 40 predicted sequence amplicons to discriminate among 317 serotypes of *S. enterica* subspecies I. We extracted the sequence for 27 genes on 317 strains of *S. enterica* subspecies I using phylogenetic analysis (Figure 1 B, Figure S1). All 40 primers also were tested in the laboratory with a limited set of isolates. Expected amplicons were generated (or not for negative controls and *E. coli*) for these limited experiments (data not provided).

All NCBI Salmonella genomes are linked to NCBI Sequence Read Archive (SRA) files, and accession numbers. Cultures included in this study are also available upon request. Please direct any queries for isolates to our strain curator Dwayne Roberson, at Dwayne.Roberson@fda.hhs.gov.

## Supporting Information

Figure S1 **Majority Rule Bootstrap consensus tree of 317** ***S. enterica*** **subspecies I serotypes MLST super-matrix composed of 12055 characters from 27 alignments derived from predicted PCR products made from the** ***S. enterica*** **subspecies 1 specific biomarker sequences in** **Table 3** **.**
(TIFF)

Figure S2 **Majority Rule Bootstrap consensus tree of a MLST super-matrix composed of 319 taxa (including 2** ***S. enterica houtenae*** **strains) 16221 characters from 40 alignments derived from the predicted PCR products made from the** ***S. enterica*** **subspecies 1 and** ***S. enterica houtenae*** **specific biomarker sequences in** **Table 3** **.**
(TIFF)

Table S1 (TXT)

Table S2 (TXT)

Table S3 (CSV)

Table S4 (CSV)

Table S5 (TXT)

Table S6 (TXT)
